# FBXL10 promotes EMT and metastasis of breast cancer cells via regulating the acetylation and transcriptional activity of SNAI1

**DOI:** 10.1038/s41420-021-00722-7

**Published:** 2021-10-30

**Authors:** Yangyang Yang, Binggong Zhao, Linlin Lv, Yuxi Yang, Shujing Li, Huijian Wu

**Affiliations:** School of Bioengineering & Key Laboratory of Protein Modification and Disease, Liaoning Province, Dalian University of Technology, Dalian, Liaoning Province China

**Keywords:** Breast cancer, Epithelial-mesenchymal transition

## Abstract

F-box and leucine-rich repeat protein 10 (FBXL10) has been reported to play a regulatory role in the initiation and development of breast cancer. Bioinformatics analyses revealed that FBXL10 may involve in the process of cytoskeleton organization. This research aimed to investigate the function of FBXL10 in epithelial-mesenchymal transition (EMT) and metastasis of breast cancer, and tried to reveal the molecular mechanism involved in this issue. Functional experiments in vitro revealed that FBXL10 promoted the migration and invasion of breast cancer cells through inhibiting E-cadherin expression and inducing EMT. Mechanical studies revealed that FBXL10 could specifically interact with SNAI1, but not Slug or ZEB1. And it promoted the transcriptional repression activity of SNAI1 on *CDH1* in breast cancer cells. Furthermore, FBXL10 had a positive role for the deacetylation of SNAI1 by facilitating the interaction between SNAI1 and HDAC1, a dominating deacetylase of SNAI1. And the deacetylated SNAI1 showed a more suppressive ability to inhibit the transcription of E-cadherin. Moreover, mouse models were also conducted to confirm the effect of FBXL10 on the lung metastasis of breast cancer in vivo. Totally, our data revealed that FBXL10 served as a pro-metastatic factor in breast cancer via repressing the expression of E-cadherin and inducing EMT. It may provide a novel regulatory axis in the EMT of breast cancer.

## Introduction

Breast cancer cells exhibit high heterogeneity and diverse differentiation levels, key gene mutations and aberrant activation of signaling pathways endow the cells with high motivation and aggression, which is the main cause for the lethality of carcinoma patients [1, 2]. High-grade breast cancer cells with embryonic stem-like phenotype show lower differentiation status and more flexible plasticity [1, 3], these characteristics are also required in transition states in tumor initiation and progression [4]. EMT, which was originally found in embryonic development and tissue remodeling, has been demonstrated to play a role in the invasion and metastasis of breast cancer [5, 6]. The process of EMT involves the induction of mesenchymal markers (N-cadherin and vimentin) and reduction of epithelial markers (E-cadherin and cytokeratin 18). And this process was also manufactured by the dynamic regulatory network of transcriptional factors (e.g., SNAI1 [7, 8], SLUG [9], ZEB1/2 [10], and Twist [11]) and hyperactivation of extracellular signaling pathways including transforming growth factor beta (TGF-β) [12], Wnt/β-catenin [7, 13, 14], and Notch [15–17]. The application of spontaneous metastatic mouse models with fluorescent lineage labeling in vivo showed that the breast cancer cells with metastatic ability were usually undergoing EMT and simultaneously coupled with E-cadherin loss or downregulation [18–20]. In addition, subpopulation of metastatic tumor cells also displayed up-regulation or hyperactivation of EMT-related transcriptional factors [18, 21]. SNAI1, known as a canonical transcription suppressor, activates the EMT program and plays a vital role in fibrosis, embryogenesis and cancer progression [7, 8, 22]. It can directly bind to E-box motif of epithelial genes promoters (e.g., *E-cadherin*) through its C‑terminal zinc‑finger domains, and then the transcription initiates through recruiting the polycomb repressive complex 2 (PRC2) which is coordinated with histone modifications (e.g., methylation and acetylation at H3K4 [23], H3K9 [24, 25], and H3K27 [26, 27]). Therefore, SNAI1 is usually regarded as one of the master transcription factors of EMT and a metastatic marker in cancer progression. Furthermore, the cellular location and transcription activity of SNAI1 are largely associated with its post-translational modifications (PTMs) [11]. For example, phosphorylation of SNAI1 at two Ser-rich motifs mediated by glycogen synthase kinase‑3β (GSK3β) inactivated its transcriptional function via inducing its nuclear export and facilitated the subsequent ubiquitin-mediated degradation [28, 29]. Acetylation of SNAI1 at K146 and K187 catalyzed by CREB-binding protein (CBP) prevented the association of SNAI1 with repressor complex [30]. And in gastric cancer, bromodomain-containing protein 4 (BRD4) recognized acetylated SNAI1 and prevented its interaction with E3 ligase FBXL14 and β-Trcp1, further decreased its polyubiquitination and proteasomal degradation [31]. However, the dynamic regulatory network of SNAI1 PTMs during EMT in breast cancer has not been fully illustrated, so more factors associated with modifications of SNAI1 should be identified in cancer progress.

FBXL10 (also named KDM2B), belongs to the F-box protein family, is a multiple functional protein which was reported to play a vital role in multiple physiological processes, such as senescence, cell proliferation, apoptosis, metabolism regulation and stem-phenotype maintaining [32–35]. FBXL10 cooperates with S-phase kinase-associated protein 1 (SKP1) and Cullins to form the SCF ubiquitin E3 ligase for specific recognition of proteins targeted for ubiquitination and degradation [36]. Furthermore, the JmjC and PHD domains of FBXL10 endow its demethylase activity to remove the H3K4me3 [37], H3K36me2 [38], and H3K79me2/3 [39, 40]. Studies demonstrated that high expression of FBXL10 was detected in many types of tumors, and its oncogenic function was closely associated with high aggression of cancers and poor prognosis of patients [41–43]. It has been reported that FBXL10 was overexpressed in breast cancer, knockdown of FBXL10 induced cell cycle arrest, decreased cell proliferation and tumorigenesis [43, 44]. In contrast, another research found that FBXL10 served as a tumor suppressor via regulating the ribosome biogenesis [45]. These results indicated that FBXL10 may play a diverse role in the development of breast cancer. Therefore, the defined role and regulatory mechanism of FBXL10 in the initiation and development of breast cancer still remains to be explored.

Herein, we explored the potential role and molecular mechanism of FBXL10 in the invasion and metastasis of breast cancer cells, and evaluated the oncogenic function of FBXL10 and its synergistic effect on SNAI1 in EMT and breast cancer progression.

## Results

### FBXL10 induced EMT and invasion of breast cancer cells

**Fig. 1:**
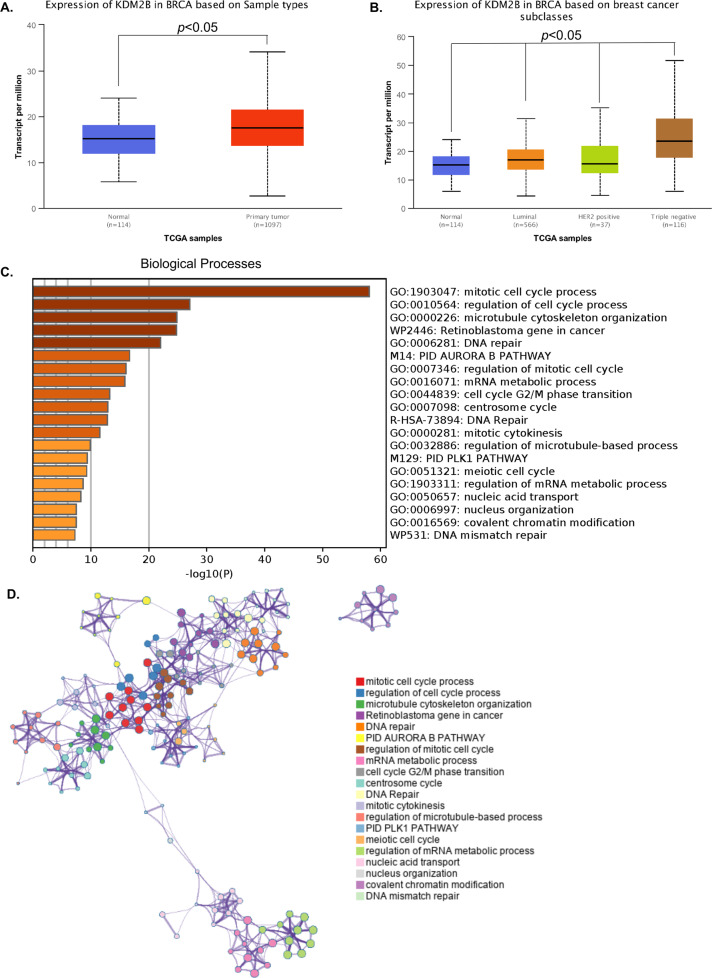
Bioinformatics analysis of FBXL10 and its associated genes in breast cancer. **A**, **B** The expression of FBXL10 in breast cancer based on sample types (**A**) and major subtype (**B**), these data were analyzed and downloaded from UALCAN database (http://ualcan.path.uab.edu/). **C**, **D**. GO enrichment analysis (**C**) and cluster analysis (**D**) of biological processes of FBXL10 positively associated genes in breast cancer. The genes cluster was downloaded from UALCAN database and GO analysis was conducted with Metascape (http://metascape.org/).

**Fig. 2:**
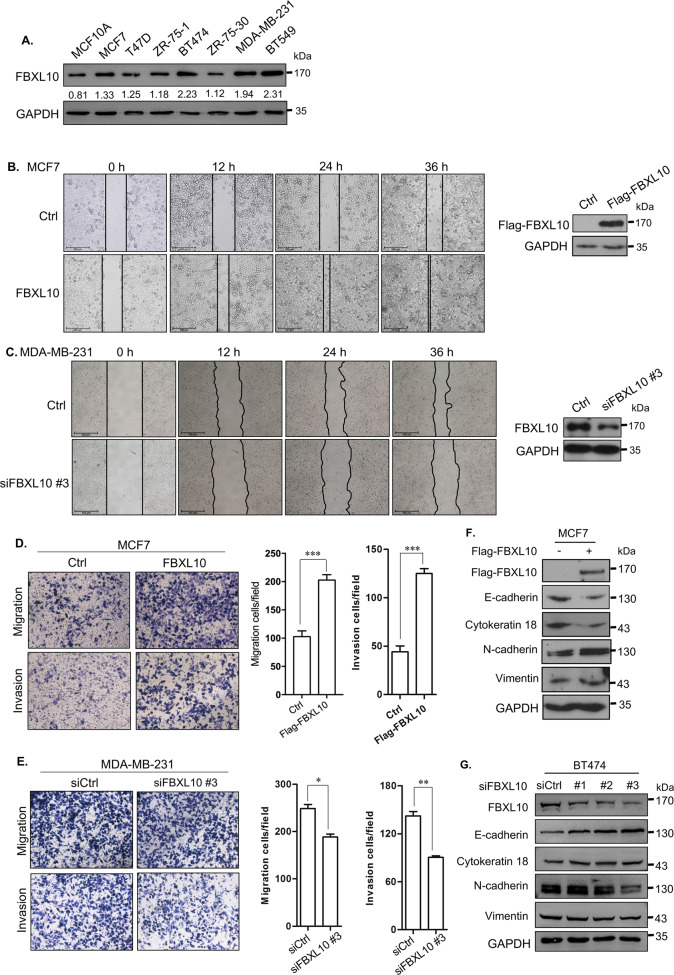
FBXL10 modulates breast cancer cells motility, invasion via regulating EMT. **A** Western blot detected the expression of FBXL10 in normal breast epithelial cell and different types of breast cancer cells. The relative expression of FBXL10 was normalized by GAPDH with gray level ratio analysis. **B**, **C** Scratch wound-healing assay evaluated the effect of FBXL10 overexpression or knockdown on the migration of MCF7 (**B**) and MDA-MB-231 (**C**) cells respectively. **D**, **E** Transwell assay assessed the effect of FBXL10 overexpression or knockdown on the migration and invasion of MCF7 (**D**) and MDA-MB-231 (**E**) cells respectively. The bar graph showed the number of migrating and invading cells for each category of cells with three independent replications. *P < 0.05; **P < 0.01; ***P < 0.001. **F** Western blot assay tested the EMT markers (E-cadherin, Cytokeratin 18, N-cadherin and Vimentin) in MCF7 cells with FBXL10 overexpression. **G** Western blot tested the effect of FBXL10 knockdown with siRNAs on the expression of EMT markers in BT474 cells.

Considering that FBXL10 was dysregulated in some types of solid tumors, bioinformatics analysis using UALCAN dataset (http://ualcan.path.uab.edu/) was conducted based on The Cancer Genome Atlas (TCGA) to determine the expression of FBXL10 in breast cancer [46]. It showed higher expression of FBXL10 in breast carcinomatous tissues compared with normal tissues (Fig. 1A), and analysis based on major subtypes of breast cancer indicated that FBXL10 exhibited the highest expression levels in triple-negative breast cancer tissues compared with other less malignant subclasses (Fig. 1B). What’s more, the functional genes positively correlated with FBXL10 (Pearson Correlation Coefficient ≥ 0.5) in breast cancer (Table S1) were analyzed in Metascape (http://metascape.org/) [47]. GO enrichment and cluster analysis of biological processes showed that the associated genes of FBXL10 not only participated in cell proliferation and cell cycle, but played a vital role in regulating cytoskeleton organization (Fig. 1C, D). The emerging literature evidence demonstrated that invasion and metastasis of breast cancer cells were coupled with cytoskeletal reconstruction, which process was referred to EMT [2]. Therefore, these results indicated FBXL10 may serve as an oncogenic factor in EMT and metastasis of breast cancer cells. We first detected the expression of FBXL10 in several breast cancer cell lines and normal breast epithelial cell. It showed that FBXL10 exhibited relative higher expression in the breast cancer cells with mesenchymal-like morphology and aggressive character (BT474, MDA-MB-231, and BT549) (Fig. 2A), this result was consistent with our bioinformatics analysis and Kottakis’s research reported previously [44]. They indicated that FBXL10 served as a poor prognostic factor in breast cancer and its expression may be associated with the aggressive potential of breast cancer cells [44]. To investigate the biological function of FBXL10 in the metastasis of breast cancer, cell scratch wound-healing and transwell assays were conducted in MCF7 and MDA-MB-231 cells. The results showed that overexpression of FBXL10 in MCF7 exhibited significantly enhanced migration and invasion ability (Fig. 2B, D). In contrast, knockdown of FBXL10 could markedly inhibit cell metastatic potential of MDA-MB-231 (Fig. 2C, E and Fig. S1A). These results indicated that FBXL10 could promote cell mobility and invasion of breast cancer cells. Previous studies demonstrated that the aggressive breast cancer cells had a stem-like phenotype and underwent EMT when metastasis occurred, and FBXL10 played a vital role in maintaining self-renewal of stem cell [44, 48]. Therefore, we hypothesized that FBXL10 may participate in EMT and further regulate metastasis of breast cancer. To verify our assumption, the epithelial and mesenchymal markers were examined by Western blot. As shown, significantly reduced expression of epithelial markers (E-cadherin and Cytokeratin 18), and increased mesenchymal markers (N-cadherin and Vimentin) were observed in MCF7 cells with FBXL10 overexpression (Fig. 2F). Knockdown of FBXL10 increased the expression of E-cadherin and Cytokeratin 18, while N-cadherin and Vimentin expression had decreasing trends (Fig. 2G). Totally, these data indicated that FBXL10 induced migration and invasion of breast cancer cells through modulating EMT.

### FBXL10 inhibited the expression of E-cadherin

**Fig. 3:**
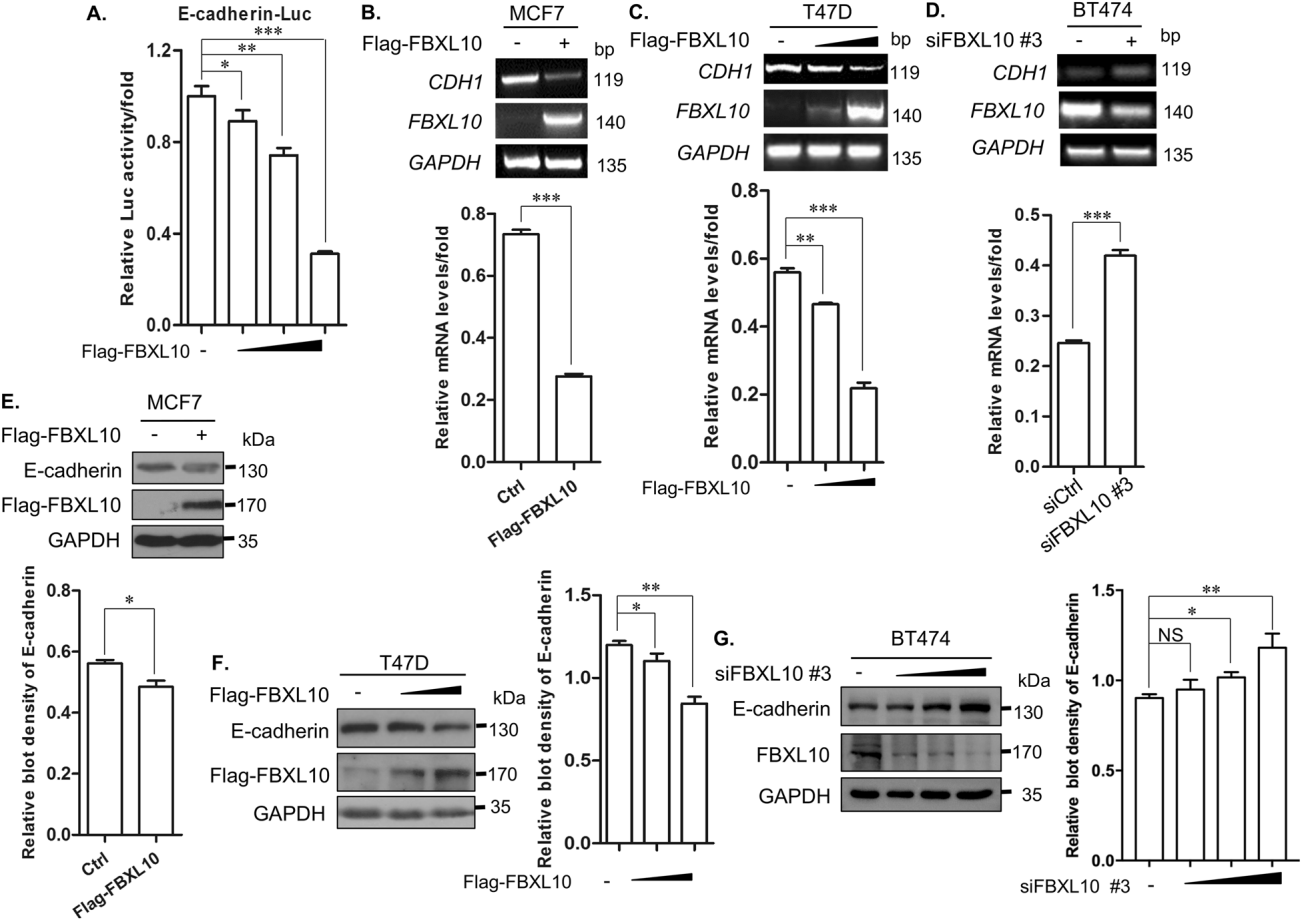
FBXL10 inhibits the expression of E-cadherin in breast cancer cells. **A** Reporter gene assay showed the effect of FBXL10 overexpression on E-cadherin luciferase activity. **B**, **C** RT-PCR assay assessed the *CDH1* mRNA levels with overexpression of FBXL10 in MCF7 (**B**) and T47D (**C**) cells. The bar graph below showed the relative intensity of *CDH1* mRNA normalized by *GAPDH*. Data are means ± SD (*n* = 3). **P < 0.01; ***P < 0.001. **D** RT-PCR assay tested the effect of FBXL10 knockdown with siRNA on *CDH1* mRNA levels in BT474 cells. The bar graph below showed the relative intensity of *CDH1* mRNA normalized by *GAPDH*. Data are means ± SD *(n* = 3). ***P < 0.001. **E**, **F** Western blot assay was performed to evaluate the E-cadherin protein levels in MCF7 (**E**) and T47D (**F**) cells with FBXL10 overexpression. The bar graph showed the relative intensity of E-cadherin protein normalized by GAPDH. Data are means ± SD (*n* = 3). *P < 0.05; **P < 0.01. **G** Western blot assay evaluated the effect of siFBXL10 on E-cadherin protein levels in BT474 cells. The bar graph showed the relative protein density of E-cadherin protein normalized by GAPDH. Data are means ± SD (*n* = 3). *P < 0.05; **P < 0.01; NS means no significant difference.

E-cadherin not only served as a cell adhesion structure molecular, but was a vital factor in maintaining the epithelial characteristic of cells. We observed that overexpression of FBXL10 could reduce the expression of E-cadherin (Fig. 2F), and bioinformatics analysis from TCGA datasets showed that the expression of *FBXL10* was negatively associated with *CDH1* in breast cancer (Fig. S1B), so it provoked our thinking that whether FBXL10 could repress the expression of E-cadherin and further modulate EMT process. Initially, the reporter gene assay revealed that FBXL10 could inhibit the transcriptional activity of E-cadherin-Luc in a dose-dependent way (Fig. 3A), which indicated that FBXL10 may directly modulate the transcription of *CDH1*. Next, the mRNA levels of *CDH1* were measured by RT-PCR in MCF7, T47D and BT474. Significantly reduced mRNA levels of *CDH1* were observed when FBXL10 was overexpressed (Fig. 3B), and the repressive effect of FBXL10 on *CDH1* transcription was also dose-dependent (Fig. 3C). In contrast, deletion of FBXL10 in BT474 enhanced the *CDH1* mRNA levels (Fig. 3D). Furthermore, the protein levels of E-cadherin were also examined in breast cancer cells, the regulatory effect of FBXL10 on E-cadherin protein levels was consistent with its mRNA (Fig. 3E–G). These results indicated that FBXL10 could repress the transcription of *CDH1*.

### FBXL10 interacted with SNAI1

**Fig. 4:**
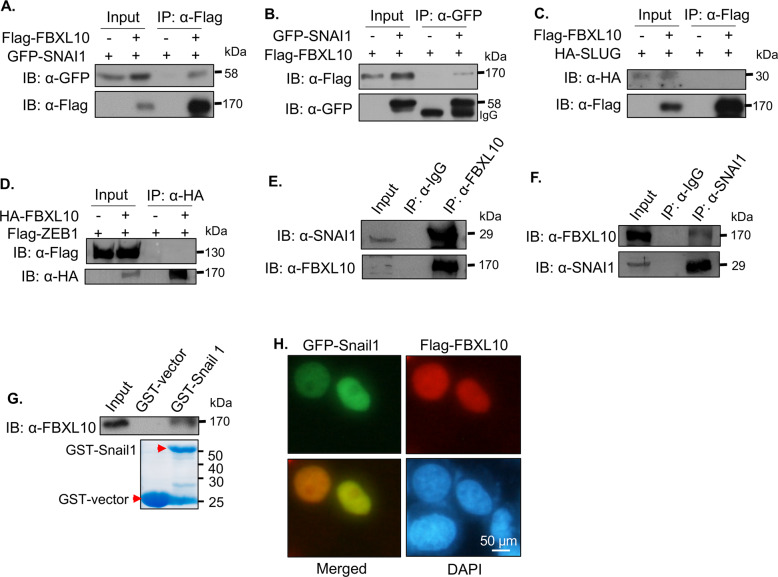
The interaction between FBXL10 and SNAI1. **A**, **B** CoIP assay showed the interaction between exogenous FBXL10 and SNAI1 in HEK293T cells. **C**, **D** CoIP assay was conducted to explore the interaction between FBXL10 with SLUG (**C**) and ZEB1 (**D**) in HEK293T cells. **E**, **F**. CoIP assay tested the endogenous interaction between FBXL10 and SNAI1 in ZR-75–30 cells. **G** GST-pulldown assay showed the purified SNAI1 had physical interaction with endogenous FBXL10 from MCF7 cells. **H** Immunofluorescence assay showed the colocalization of FBXL10 (red) and SNAI1 (green) in MCF7 cells.

To further investigate the underlying mechanism of FBXL10 in modulating the expression of E-cadherin, the well-known transcriptional repressors of *CDH1* were introduced together with FBXL10 in the reporter gene assay, it revealed that FBXL10 could enhance the repression role of the three transcriptional repressors (SNAI1, SLUG, and ZEB1) on E-cadherin-Luc activity (Fig. S1C). It suggested that FBXL10 may inhibit the transcription of *CDH1* via certain classical transcriptional repressors. To verify this possibility, CoIP assays were performed to determine the interaction between them. The results showed that the exogenous protein interaction could be detected between FBXL10 with SNAI1 (Fig. 4A, B), but not with SLUG (Fig. 4C) and ZEB1 (Fig. 4D). Moreover, the association between endogenous FBXL10 and SNAI1 was also confirmed in the breast cancer cells (Fig. 4E, F). And GST-pulldown assay also revealed that the purified SNAI1 could physically interact with endogenous FBXL10 from MCF7 cells (Fig. 4G), and the immunofluorescence assay revealed that FBXL10 and SNAI1 were colocalized in the nucleus in MCF7 cells (Fig. 4H). Taken together, these results revealed that FBXL10 physically interacted with SNAI1 in breast cancer cells, but not SLUG or ZEB1.

### FBXL10 enhanced the repressive effect of SNAI1 on *CDH1* transcription

**Fig. 5:**
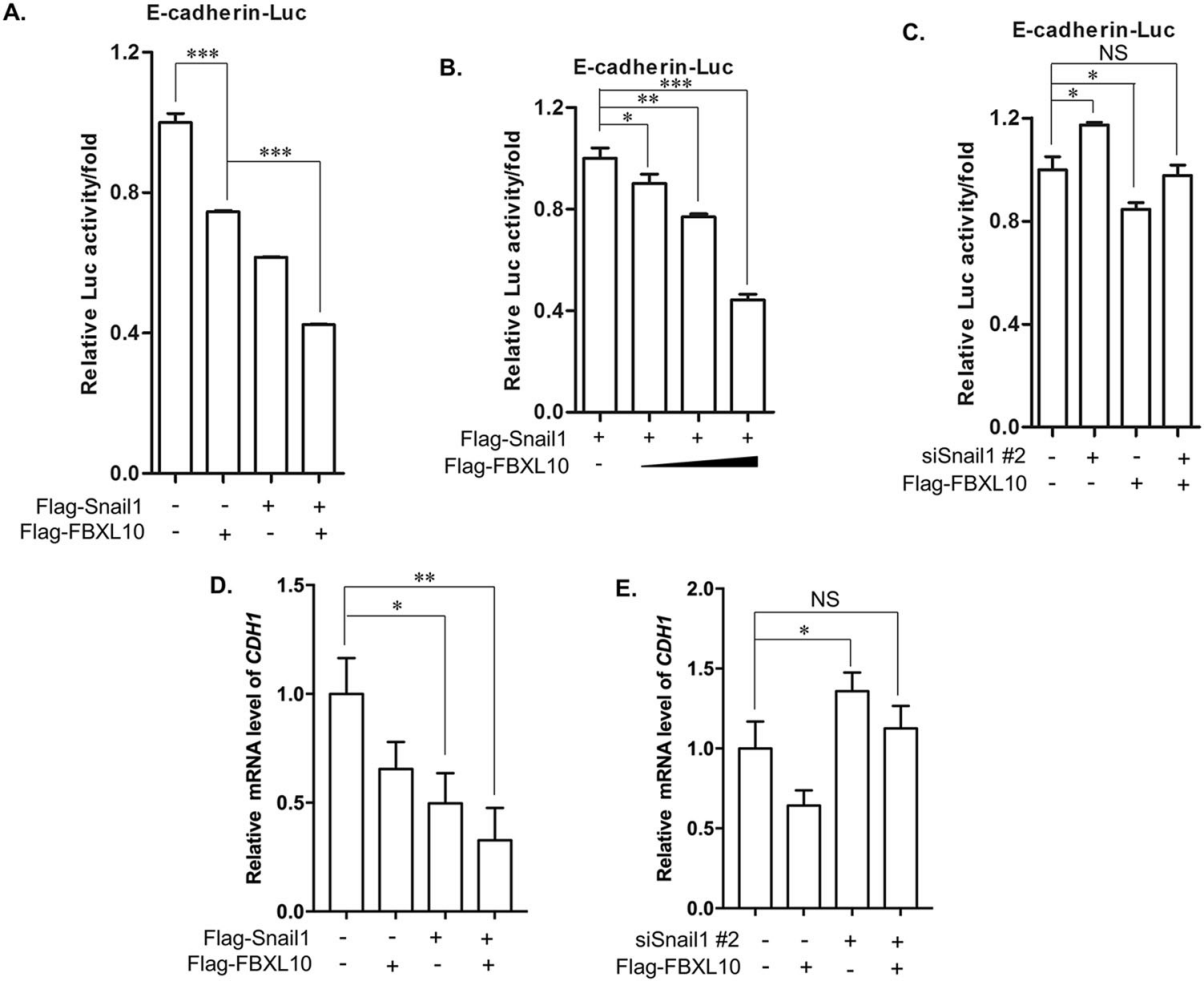
FBXL10 enhanced the trans-repression of SNAI1 on *CDH1*. **A**, **B**. Reporter gene assay showed the regulation of E-cadherin luciferase activity by FBXL10 and SNAI1 overexpression. *P < 0.05; **P < 0.01; ***P < 0.001. **C** Reporter gene assay showed the effect of FBXL10 cooperated with or without siSNAI1 on the E-cadherin luciferase activity. **D**, **E**. qPCR assays assessed the effect of FBXL10 and SNAI1 on *CDH1* mRNA levels in MCF7 cells.

To further confirm whether FBXL10 could modulate *CDH1* expression via SNAI1, the reporter gene assay showed that the overexpression of SNAI1 or FBXL10 alone could both significantly inhibit the E-cadherin-Luc activity, and the addition of SNAI1 and FBXL10 together showed more obviously repressive effect (Fig. 5A). And it also showed that the repressive effect of SNAI1 on E-cadherin-Luc activity was dose-dependent with the addition of FBXL10 (Fig. 5B). Moreover, the regulation of FBXL10 on E-cadherin-Luc activity was partially dependent on SNAI1, as the repressive role of FBXL10 on the E-cadherin-Luc was rescued with SNAI1 knockdown (Fig. 5C, S1D). Additionally, qPCR assays revealed that the regulatory effect of FBXL10 and SNAI1 on the *CDH1* mRNA levels was consistent with the results of reporter gene assays (Fig. 5D, E). In conclusion, these results indicated that FBXL10 enhanced the transcriptional repression of SNAI1 on E-cadherin expression.

### FBXL10 enhanced the interaction between SNAI1 with HDAC1 and facilitated HDAC1-mediated deacetylation of SNAI1

**Fig. 6:**
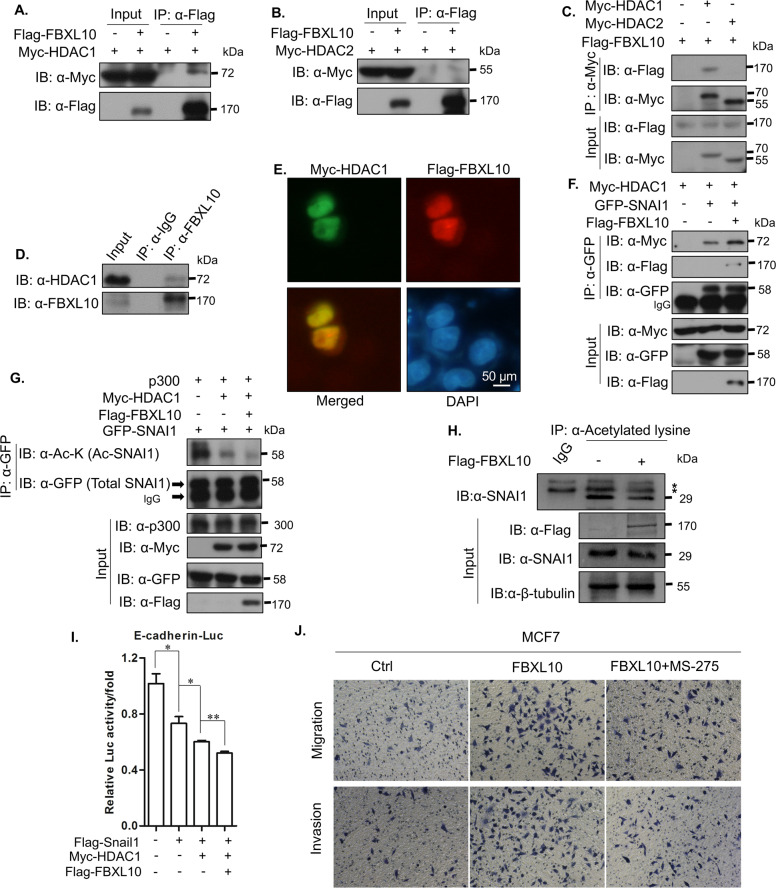
FBXL10 enhanced the interaction between SNAI1 with HDAC1 and facilitated HDAC1-mediated deacetylation of SNAI1. **A**–**C** CoIP assay showed the association between exogenous FBXL10 with HDAC1 and HDAC2. **D** CoIP assay showed the endogenous protein interaction between FBXL10 and HDAC1. **E**. Immunofluorescence assay showed the colocalization of FBXL10 (red) and HDAC1 (green) in MCF7 cells. **F** CoIP assay showed the effect of FBXL10 overexpression on the interaction between SNAI1 and HDAC1. **G** CoIP assay showed the effect of FBXL10 on HDAC1-mediated deacetylation of exogenous SNAI1. **H** CoIP assay showed the effect of overexpression FBXL10 on endogenous SNAI1 acetylation. **I** Reporter gene assay assessed the effect of FBXL10/SNAI1/HDAC1 on the E-cadherin luciferase activity. **J** Transwell assay was performed to evaluate the effect of FBXL10 and HDAC1 inhibitor MS-275(1 μM for 12 h) on the migration and invasion of MCF7 cells.

To explore the defined mechanism of FBXL10 and SNAI1 on the regulation of *CDH1*, the effect of FBXL10 on SNAI1 expression was determined by qPCR and Western blot assays. The results showed that the mRNA and protein levels of SNAI1 were not significantly affected by FBXL10 overexpression (Fig. S1E, F). As we know, the repression of SNAI1 on *CDH1* transcription was largely dependent on its co-repressor HDAC1. And recent study demonstrated that SNAI1 could be acetylated by p300/CBP and deacetylated by HDAC1/2 [30]. The acetylation of SNAI1 could inhibit the formation of its repressor complex, HDAC1/2 acted as the deacetylase of SNAI1 to decrease its acetylation and further accelerate its transcriptional repressive role [30]. As FBXL10 could enhance the repression of SNAI1 on E-cadherin expression, we sought to determine whether FBXL10 could affect the interaction between SNAI1 and HDAC1 or HDAC2. To confirm this possibility, the interaction between FBXL10 and HDAC1/2 was determined by CoIP (Fig. 6A–D), and the results revealed that FBXL10 could interact with HDAC1, but not HDAC2. And the immunofluorescence assays also confirmed their co-location in nucleus (Fig. 6E). Moreover, the CoIP assay revealed that overexpressed FBXL10 could markedly promote the interaction between HDAC1 and SNAI1 (Fig. 6F). This reminded us that whether FBXL10 could modulate the acetylation of SNAI1 via HDAC1. To investigate this notion, the acetylation of SNAI1 was detected with the treatment of acetyltransferase p300. As shown, HDAC1 could significantly reduce the acetylation of SNAI1, and the addition of FBXL10 enhanced the HDAC1-mediated deacetylation of SNAI1 (Fig. 6G). Moreover, the endogenous SNAI1 acetylation levels were also decreased under overexpression of FBXL10 (Fig. 6H). The reporter gene assay also demonstrated that HDAC1 and SNAI1 showed more inhibitory effect on the activity of E-cadherin-Luc, and FBXL10 could enhance this effect (Fig. 6I). The specific HDAC1 inhibitor MS-275 was introduced to further evaluate whether the function of FBXL10 was dependent on HDAC1. The results showed that inhibition of HDAC1 could partially impair the effect of FBXL10 on the metastatic ability of MCF7 cells (Fig. 6J). Totally, these results revealed that FBXL10 could accelerate the recruitment of HDAC1 to SNAI1 and facilitate HDAC1-mediated deacetylation of SNAI1, accompanied with increased SNAI1 transcriptional repressive role on *CDH1*.

### FBXL10 enhanced the metastasis of breast cancer cells in vivo

**Fig. 7:**
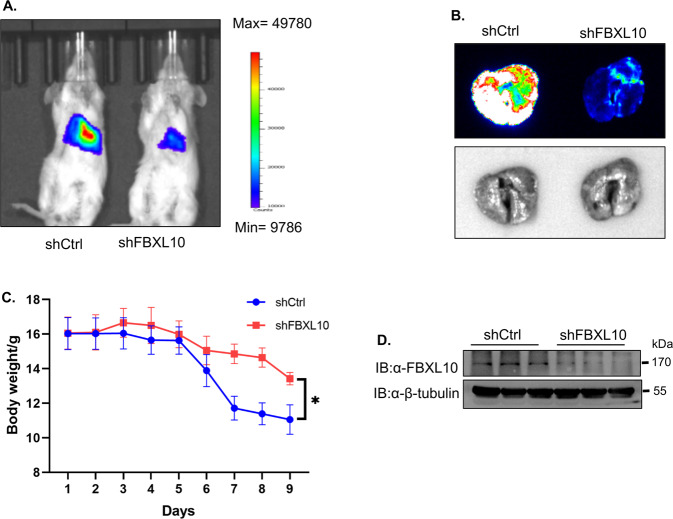
FBXL10 enhanced the metastasis of breast cancer cells in mouse model. **A** Quantitative bioluminescence imaging showed the effect of shFBXL10 on the metastasis potential of 4T1-luc cells in BALB/C mice. **B** Bioluminescence imaging showed the relative metastasis potential of 4T1-luc cells in lung of BALB/C mice. **C** Line bar image showed the body weight of BALB/C mice injected with 4T1-luc cells stably transfected with shCtrl or shFBXL10. **D** Western blot showed the expression levels of FBXL10 in the 4T1-luc transfected with shCtrl or shFBXL10.

As E-cadherin is necessary for lung metastasis in breast cancer cells [20], and FBXL10 significantly repressed the E-cadherin transcription, the role of FBXL10 on metastasis of breast cancer was evaluated in vivo by using the mouse model. Murine basal-like breast cancer cells 4T1 with stable Luciferase expression were intravenously injected into BALB/C mice to simulate the metastasis of breast cancer cells in circulation. As expected, quantitative bioluminescence imaging showed that the mice injected with 4T1-Luc cells transfected with shFXBL10 showed less metastatic event in lung compared with the control group (Fig. 7A). The images of lungs that were obtained after the mouse was sacrificed also showed lower bioluminescence and metastases (Fig. 7B). And the group treated with shFBXL10 exhibited less body loss (Fig. 7C). In addition, the Western blot assay verified the effective knockdown of FBXL10 with shFBXL10 in 4T1-luc cells (Fig. 7D). In conclusion, these results demonstrated that FBXL10 served as a pro-metastatic factor of breast cancer cells in vivo.

## Discussion

FBXL10 has been reported to be overexpressed in gynecological tumors, but its role in tumorigenesis and progression was still controversial [41, 43, 44]. Kottakis et al. and our previous research demonstrated that FBXL10 served as an oncogene to promote the self-renewal and proliferation of breast cancer cells via regulating multiple factors including miRNAs/PRC1/PRC2 axis and estrogen-related receptor α (ERRα)/peroxisome proliferator-activated receptor gamma coactivator 1-beta (PGC1-β) signaling [43, 44]. Additional, genomics functional screening found that FBXL10 was also associated with anti-estrogen resistance in breast cancer [49]. Nevertheless, another study revealed that FBXL10 knockdown triggered a more aggressive phenotype in breast cancer, and mechanism investigation demonstrated that FBXL10 acted as a tumor suppressor through regulating ribosome biogenesis and protein synthesis [45]. Therefore, the definite function of FBXL10 in the initiation and development of breast cancer needs more research to be fully illustrated. In this study, we explored a novel mechanism of FBXL10 involved in the invasion and metastasis of breast cancer. Firstly, we revealed that FBXL10 enhanced the migration and invasion of breast cancer cells via regulating EMT, which plays vital role in the metastasis of breast cancer. Secondly, we found that FBXL10 decreased the E-cadherin transcription expression by enhancing the repressive function of SNAI1. Thirdly, molecular mechanism investigation revealed that FBXL10 physically interacted with SNAI1 and decreased its acetylation via promoting the interaction between SNAI1 and HDAC1. Finally, mice model was used to evaluate the role of FBXL10 on metastasis of breast cancer cells in vivo, knockdown of FBXL10 significantly suppressed the lung metastasis of breast cancer. Totally, this study highlighted the function of FBXL10 in EMT and the metastasis of breast cancer, which may contribute novel insight of FBXL10 in the development of breast cancer.

EMT is a dynamic cellular process with a change of cell morphology and function [5, 50]. During EMT, cell conjunction and cell-extracellular matrix interactions of epithelial-like cells are reprogrammed, which induces the loss of cell-cell adhesion and detachment of cellular basal membrane, and accompanied with novel transcription regulation activation to drive the mesenchymal phenotype [23]. Through the research of EMT in many types of tumors, the conversion from epithelial-like to mesenchymal-like was always not complete and continued [4]. The incomplete transition was referred to partial EMT, subpopulation of mammary tumor cells displaying different degree of epithelial and mesenchymal phenotypes, which was termed as mixed or hybrid EMT [4]. And the mix or hybrid EMT was essential for tumorigenesis of basal-like breast cancer [51]. Although our study revealed a positive role of FBXL10 in EMT, whether this progress was intended to complete EMT or hybrid EMT was still unclear. Therefore, the single-cell technology or fluorescence-labeled cells with real-time detection system were required to answer this question in the future.

FBXL10 could also function as a histone demethylase, and remove the H3K4me3, H3K36me2 and H3K79me2/3 of CpG island to regulate the chromatin conformation [37–40]. The regulation of E-cadherin transcription was largely affected by promoter methylation, heterogeneous E-cadherin promoter methylation was found in the early and invasive stage of breast cancer [52]. Whether FBXL10 could bind to CpG island of E-cadherin promoter and affect its gene expression through methylation regulation, this issue also needs to be explored in follow-up research. In addition, the initiation and development of EMT are mediated by complex signaling crosstalks, whether FBXL10 could play a role in canonical signaling pathways involved in EMT such as TGF-β [12] and Wnt/β-catenin [13], this question is also deserved investigation. Our study and other study showed that FBXL10 was overexpressed in breast cancer, the upstream factors or signals that triggered the higher expression of FBXL10 in mammary tumors still need to be addressed. E-cadherin served as a cell adhesion structure molecular and a mediator of cellular signaling transduction, the expression of E-cadherin was not only dependent on the transcriptional regulation, but also its post-translational modifications, including glycosylation [53], phosphorylation [54] and ubiquitination [55]. Zhou et al. showed that N-glycosylation of E-cadherin at Asn-637 was essential for its expression, folding and trafficking [53, 55]. Ubiquitination of E-cadherin mediated by SCF complex containing SKP2 or CBLL1/HAKAI directly triggered its proteasome degradation, which process required prior phosphorylation of E-cadherin by CK1/CSNK1A1 [54]. FBXL10 cooperates with SKP1 and forms the SCF ubiquitin E3 ligase to mediate protein degradation [36], whether FBXL10 could collaborate with SKP1 or SKP2 to regulate the ubiquitination of E-cadherin remains to be explored.

Totally, we explored a potential mechanism that FBXL10/SNAI1/HDAC1 axis promoted EMT and metastasis of breast cancer cells. The specific interaction between FBXL10 and SNAI1 enhanced the repressive role of SNAI1 on *CDH1* by facilitating HDAC1-mediated deacetylation of SNAI1. This present evidence may contribute a better understanding of FBXL10 and SNAI1 in metastasis of breast cancer, it may provide a novel therapeutic approach to develop the targeted drugs or inhibitors for the treatment of breast cancer.

## Materials and methods

### Cell culture

Human embryonic kidney HEK293T, and breast cancer cell lines MCF7, T47D, ZR-75-1, ZR-75-30 and MDA-MB-231 were previously described in our published studies [43, 56]. MCF7 were cultured in Eagle’s Minimum Essential Medium (EMEM) containing 10% fetal bovine serum, 1% penicillin-streptomycin and 0.01 mg/ml human recombinant insulin. T47D were cultured in complete RPMI-1640 medium in addition to 2 Units/mL bovine insulin. ZR-75-1 and ZR-75-30 were cultured in RPMI-1640 medium containing 10% fetal bovine serum and 1% penicillin-streptomycin. BT474 and BT549 were kindly provided by Dr. Zhaowei Xu of Binzhou Medical University and cultured in RPMI-1640 completed culture. Murine basal-like breast cancer cell 4T1 stably expressed Luciferase was cultured in RPMI-1640 with 10% fetal bovine serum [57]. Breast normal epithelial cell MCF10A was cultured in DMEM/F12 medium containing 5% horse serum, 20 ng/ml EGF, 0.5 μg/ml hydrocortisone, 100 ng/ml cholera toxin, 10 μg/ml insulin and 1% penicillin-streptomycin. All of the cell lines were maintained at 37 °C in humid incubator with 5% CO_2_.

### Antibodies and reagents

FBXL10 rabbit polyclonal antibody (09–864) was purchased from Merck Millipore. Rabbit monoclonal antibodies against SNAI1 (3879), E-cadherin (3195), N-cadherin (13116), Vimentin (5741) and acetylated-lysine (9441) were obtained from Cell Signaling Technology. Rabbit monoclonal antibody against Cytokeratin 18 (ab133272) was obtained from Abcam. GAPDH (sc-47724) and rabbit polyclonal p300 (sc-585) were purchased from Santa Cruz Biotechnology. Flag (F7425) and MYC (SAB5700727) antibodies were purchased from Sigma. GFP (GTX113617) and HA (GTX115044) were obtained from GeneTex. Protein A/G and Flag-affinity magnetic beads were purchased from Biomake. GFP-selector (2-9131-020) beads were obtained from IBA Life Sciences. MS-275 (S1503) was purchased from Selleck.

### Plasmids and transfection

The human FBXL10 expression plasmid was used as previously described [43]. The GFP-SNAI1, HA-SLUG, Flag-ZEB1, Myc-HDAC1 and Myc-HDAC2 were constructed from our lab [58]. p300 plasmid was mentioned in our previous study [56]. For HEK293T, polyetherimide was used for transfection reagent. Breast cancer cells were transfected with jetPRIME transfection reagent (Polyplus, 114-15) according to the manufacturer’s specifications. The siRNAs for human *FBXL10* were obtained from Hippobiotec, the sequences were listed: 5′-GCAAACAGAGUGACAUCUUTT-3′ (#1 sense), 5′-AAGAUGUCACUCUGUUUGCCT-3′ (#1 antisense); 5′-GGACCCAUCUCACUGAGUUTT-3′ (#2 sense), 5′-AACUCAGUGAGAUGGGUCCAT-3′ (#2 antisense); 5′-CCAUCUGCAAUGAAAUCAUTT-3′ (#3 sense), 5′-AUGAUUUCAUUGCAGAUGGAG-3′ (#3 antisense). The sequences of siRNAs targeted for *SNAI1* were listed: 5′-CCACUCAGAUGUCAAGAAGUA-3′ (#1 sense), 5′- UACUUCUUGACAUCUGAGUGG-3′ (#1 antisense); 5′-CCAAUCGGAAGCCUAACUACA-3′ (#2 sense), 5′-UGUAGUUAGGCUUCCGAUUGG-3′ (#2 antisense). The targeted sequence of shRNA for mouse *FBXL10* was 5′-GCTGTGGAAATATCTGTCATA-3′, shRNAs were constructed using pRNAT-U6.1/Hygro.

### CoIP, GST-pulldown, and Western blot

CoIP and Western blot were performed as described in the previous study [43]. GST-SNAI1 plasmid was constructed with pGEX-4T-2, the GST-SNAI1 purification was carried out as described previously [58]. The GST-pulldown was performed using Pierce® GST Spin Purification Kit (ThermoFisher, 16106) according to the manufacture instruction. Cells were lysed with the RIPA lysis buffer (50 mM Tris-HCl [pH 7.4], 150 mM NaCl, 0.1% SDS, 1% NP-40, 0.5% sodium deoxycholate and protease inhibitor cocktail (Biomake)). After centrifugation at 12, 000 g for 15 min at 4 °C, the supernatant was incubated with the indicated antibody or with control IgG and Protein A/G at 4 °C for 4 h. After centrifugation at 3,000 g for 10 min at 4 °C, the supernatant was abandoned and the precipitate was subjected to wash three times with washing buffer. Then the pellets were suspended with protein 2 × loading buffer, boiling at 100 °C for 5 min and subjected to SDS-PAGE. After electrophoresis, proteins were separated and blotted onto a PVDF membrane (Millipore). Membranes were probed with the specific primary antibody and then peroxidase-conjugated secondary antibodies. The bands were visualized by chemiluminescence.

### Immunofluorescence

Immunofluorescence assay was performed as described previously [43, 57, 58]. The cells transfected with GFP-SNAI1, Flag-FBXL10 and Myc-HDAC1 were seeded into 35 mm dishes with glass-bottom. After being fixed with 4% formaldehyde, Flag-tagged (1:200) and Myc-tagged (1:200) antibodies were incubated against the Flag-FBXL10 and Myc-HDAC1 respectively. The nucleus was stained with DAPI. The images shown were all captured with 40 × magnification.

### Transwell and scratch wound-healing assays

Transwell and scratch wound-healing assays were carried out as our previous study reported [57]. In order to eliminate the effect of FBXL10 on the proliferation of breast cancer, the cells were subjected to starvation for 24 h with a culture medium containing 2% FBS, and then harvested to evaluate the metastatic potential. 1 × 10^5^ cells were seeded into 6-well plate to perform the scratch wounding-healing assays with 2% FBS supplemented culture medium. The apical chambers for invasion evaluation of transwell were pre-packaged with Matrigel (Corning BioInc, USA), 4 × 10^4^ and 8 × 10^4^ cells/100 μl were seeded in transwell apical chamber with minimum medium for migration and invasion assays respectively, and basolateral chambers were incubated with complete culture medium. The images were captured at indicated times after crystal violet staining.

### Luciferase reporter gene assay

The luciferase reporter gene assay was performed as previously described [43]. Cells transfected with the appropriate plasmids were harvested with lysis buffer and the relative luciferase activity was measured by the Centro LB 960. β-galactosidase was used to normalize the relative activity.

### RNA isolation and real-time PCR

RNAios Plus (Takara, 9109) was used for RNA isolation, the procedure was performed as described previously [43]. The PrimeScript™ RT Master Mix (Takara, RR036A) and TB Green® Premix Ex Taq™ II (Takara, RR820A) were used for reverse transcription and qPCR reaction respectively. The specific primers for real-time PCR were listed: *GAPDH*, 5′-GGGTTGAACCATGAGAAGT-3′ (Forward), 5′-GACTGTGGTCATGAGTCCT-3′(reverse); *CDH1*, 5′-GGGTGTCGAGGGAAAAATAGG-3′ (Forward), 5′-CGAGAGCTACACGTTCACGG-3′(reverse); *FBXL10*, 5′- AGCCGGTCACCCATCTATGAA-3′ (Forward), 5′- CTTCTGTGTTCTCTCGAATGGTC-3′(reverse); *SNAI1*, 5′-GGGTTGAACCATGAGAAGT-3′ (Forward), 5′-GCACTGGTACTTCTTGACATCTG-3′(reverse). *GAPDH* was set as a reference gene for the normalization of targeted genes.

### Mice metastasis model and quantitative bioluminescence imaging

In vivo mice metastasis model was constructed as previously described [58–60]. Briefly, mice were randomly allocated to two groups according to body weight, each group had 5 units. 1 × 10^6^ 4T1-Luc murine breast cancer cells in 100 μl PBS buffer were injected into the tail vein of the 5–6 weeks female BALB/c mice. The body weights of each group were monitored every day after injection. On the 9th day, firefly luciferase bioluminescence signals of the 4T1/Luc cells were analyzed. Intraperitoneal injection of D-luciferin (BD Pharmingen, 556888) solution in D-PBS (Solarbio, D1040) buffer at 150 mg Luciferin/kg body weight for bioluminescence imaging using IVIS^®^Spectrum CT imaging system (PerkinElmer). Then, the mice were sacrificed under anesthesia and the lungs were removed surgically for bioluminescence imaging. All the animal experiments were performed according to the regulation set by the Ethics Committee for Biology and Medical Science of Dalian University of Technology.

### Statistical analysis

GraphPad Prism 8 software was used for data analyse and data were presented as the mean ± standard error bar of mean (SEM) or the mean ± standard deviation (SD). Each experiment was independently conducted at least three times. Two-tailed t-test to evaluate the statistical significance. Significance was expressed as a *P*-value, differences were considered significant when *p* < 0.05. *p < 0.05; **p < 0.01; ***p < 0.001; and non-significant differences were presented as NS.

## Supplementary information

Figure S1

Supplementary table 1

Supplementary materials

Related Manuscript File

## Data Availability

The datasets used and/or analyzed during the current study are available from the corresponding author on reasonable request.
