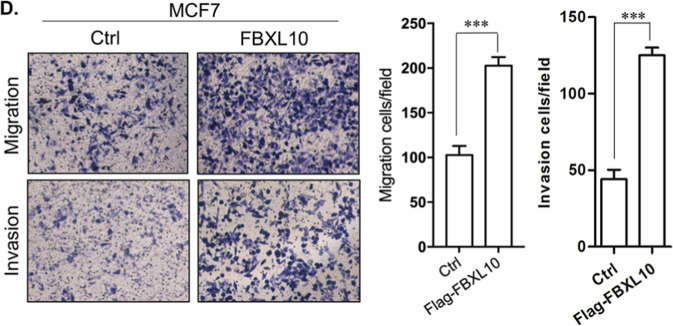# Author Correction: FBXL10 promotes EMT and metastasis of breast cancer cells via regulating the acetylation and transcriptional activity of SNAI1

**DOI:** 10.1038/s41420-022-01265-1

**Published:** 2022-12-05

**Authors:** Yangyang Yang, Binggong Zhao, Linlin Lv, Yuxi Yang, Shujing Li, Huijian Wu

**Affiliations:** School of Bioengineering & Key Laboratory of Protein Modification and Disease, Liaoning Province, Dalian University of Technology, Dalian, Liaoning Province China

**Keywords:** Breast cancer, Epithelial-mesenchymal transition

Correction to: *Cell Death Discovery* 10.1038/s41420-021-00722-7, published online 30 October 2021

Since the online publication of this article, the authors have noticed that there was an error in Fig. 2D, which arose during the assembly of the figure; specifically, the upper-left picture in Fig. 2D, showing the result of the “control of migration for MCF7” was mistakenly used. The revised version of Fig. 2D including the corrected data for “control of migration for MCF7”, was shown in the below, and the new bar graph was also provided. The authors confirmed that this mistake did not influence the data, discussion, or conclusions of this study. And we are grateful to the Editor of *Cell Death Discovery* for giving our opportunity to publish the Corrigendum, and all the authors agreed with this correction. The authors apologized for any inconvenience caused by this error.