# Supplementary figures and images for: FBXL10 promotes EMT and metastasis of breast cancer cells via regulating the acetylation and transcriptional activity of SNAI1
Source: Cell Death Discov. 2021 Oct 30;7:328. doi: 10.1038/s41420-021-00722-7 (PMC8557203; doi:10.1038/s41420-021-00722-7)

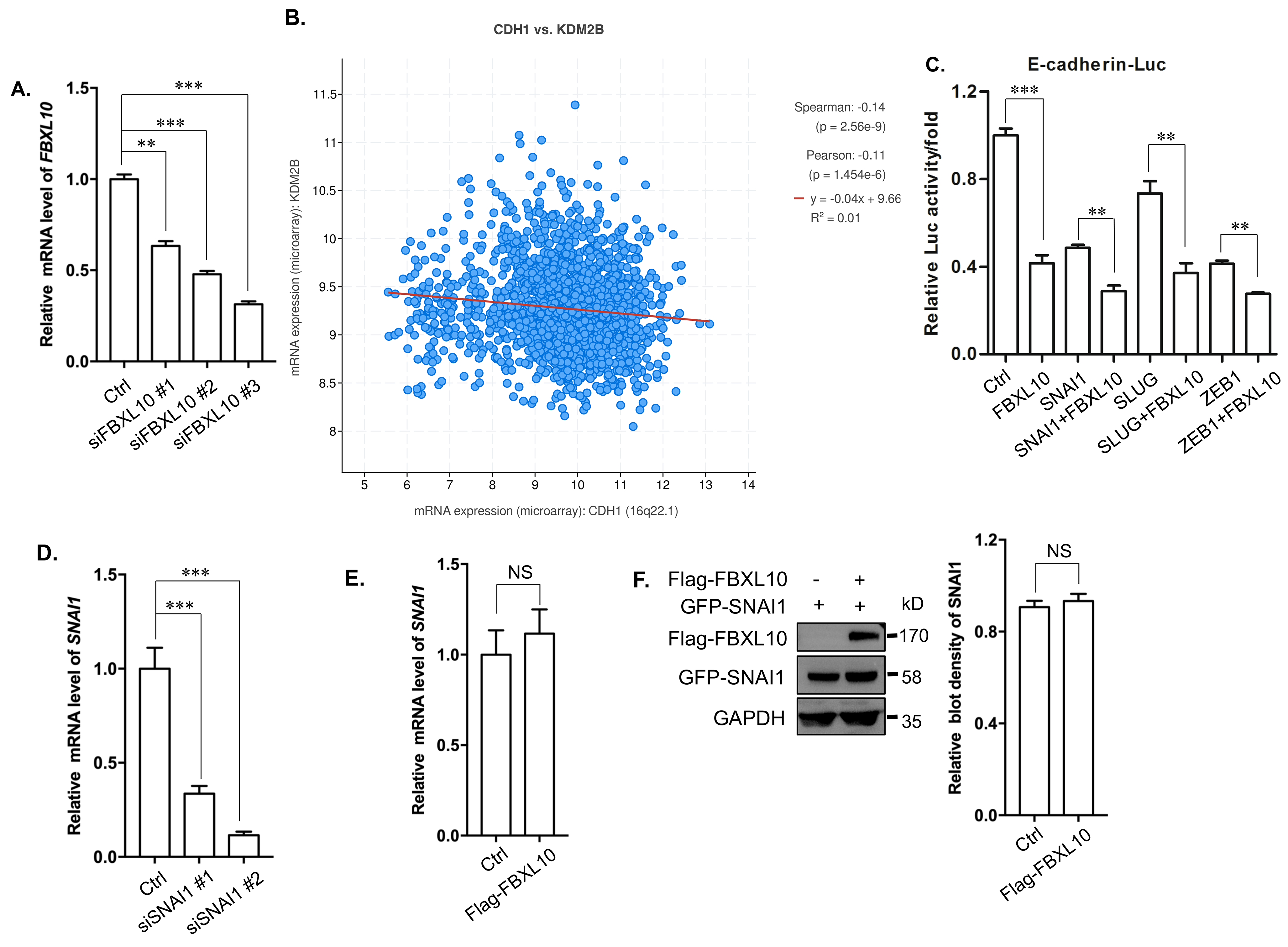

Supplement: Supplementary file 1 — Figure S1 [file 41420_2021_722_MOESM1_ESM.tif]
