# Supplementary material for: FBXL10 promotes EMT and metastasis of breast cancer cells via regulating the acetylation and transcriptional activity of SNAI1
Source: Cell Death Discov. 2021 Oct 30;7:328. doi: 10.1038/s41420-021-00722-7 (PMC8557203; doi:10.1038/s41420-021-00722-7)
Supplement: Supplementary file 3 — Supplementary materials [file 41420_2021_722_MOESM3_ESM.docx]

**Supplementary figure legends**

**Supplementary Figure 1 A.** qPCR assay assessed the silence efficacy of the three siRNAs targeting FBXL10 in MCF7 cells. **B.** TCGA BRCA dataset was used to analyze the association between *FBXL10* and *CDH1*. **C.** qPCR assay assessed the silence efficacy of the two siRNAs targeting SNAI1 in MCF7 cells. **D.** Luciferase reporter genes showed the effect of FBXL10 cooperated with factors (SNAI1, SLUG or ZEB1) on the activity of E-cadherin luciferase, ***P* < 0.01; ****P* < 0.001. **E.** qPCR assay showed the effect of overexpression FBXL10 on *SNAI1* mRNA levels in MCF7 cells. **F.** Western blot assay showed the effect of overexpression FBXL10 on endogenous SNAI1 protein levels in MCF7 cells.

**Supplementary table legends**

**Supplementary Table 1**. KDM2B/FBXL10 associated genes in breast cancer. Sheet 1. The genes positively associated with KDM2B/FBXL10 in breast cancer. Sheet 2. The genes negatively associated with KDM2B/FBXL10 in breast cancer.
