# Supplementary material for: FBXL10 promotes EMT and metastasis of breast cancer cells via regulating the acetylation and transcriptional activity of SNAI1
Source: Cell Death Discov. 2021 Oct 30;7:328. doi: 10.1038/s41420-021-00722-7 (PMC8557203; doi:10.1038/s41420-021-00722-7)
Supplement: Supplementary file 4 — Related Manuscript File [file 41420_2021_722_MOESM4_ESM.docx]

**Author contributions**

Yangyang Yang and Huijian Wu conceived and designed the study. Yangyang Yang performed the experiments and completed the original draft. Binggong Zhao, Linlin Lv, Yanan Li, Yuxi Yang and Shujing Li made formal analysis, data collection and revised the manuscript. Yangyang Yang, Shujing Li and Huijian Wu reviewed and revised the paper. All the authors have read and approved the final manuscript.
